# The influence of orthodontic fixed appliances on the oral microbiota: A systematic review

**DOI:** 10.1590/2176-9451.19.2.046-055.oar

**Published:** 2014

**Authors:** Amanda Osório Ayres de Freitas, Mariana Marquezan, Matilde da Cunha Gonçalves Nojima, Daniela Sales Alviano, Lucianne Cople Maia

**Affiliations:** 1 Doctorate student of Dentistry, Federal University of Rio de Janeiro (UFRJ); 2 Phd in Orthodontics, UFRJ; 3 Postdoc in Orthodontics, Case Western Reserve University; 4 Postdoc in Microbiology and Immunology, UFRJ; 5 Phd in Social Dentistry, Fluminense Federal University (UFF)

**Keywords:** Orthodontic appliances, Periodontics, Attachment sites, Microbiological, Microbiological analysis

## Abstract

**Objective:**

To investigate whether there is scientific evidence to support the hypothesis that the presence of orthodontic fixed appliances influences the oral microbiota.

**Methods:**

The search for articles was conducted in PubMed; ISI Web of Knowledge and Ovid databases, including articles published in English until May 17^th^, 2012. They should report human observational studies presenting the following keywords: "fixed orthodontic appliance" AND "microbiological colonization"; OR "periodontal pathogens"; OR "*Streptococcus*"; OR "*Lactobacillus*"; OR "*Candida*"; OR "*Tannerella forsythia*"; OR "*Treponema denticola*"; OR "*Fusobacterium nucleatum*"; OR "*Actimomyces actinomycetemcomitans*"; OR "*Prevotella intermedia*", OR "*Prevotella nigrescens*"; OR "*Porphyromonas gingivalis*". Articles were previously selected by title and abstract. Articles that met the inclusion criteria were analyzed and classified as having low, moderate or high methodology quality. A new detailed checklist for quality assessment was developed based on the information required for applicable data extraction for reviews. The study design, sample, follow-up period, collection and microbial analysis methods, statistical treatment, results and discussion were assessed.

**Results:**

The initial search retrieved 305 articles of which 33 articles were selected by title and abstract. After full-text reading, 8 articles met the inclusion criteria, out of which 4 articles were classified as having low and 4 as moderate methodological quality. The moderate methodological quality studies were included in the systematic review.

**Conclusions:**

The literature revealed moderate evidence that the presence of fixed appliances influences the quantity and quality of oral microbiota.

## Abstract (pt)

**Objetivo:**

investigar se há evidências científicas para comprovar a hipótese de que a presença de aparelhos ortodônticos fixos influencia a microbiota bucal.

**Métodos:**

a busca por artigos foi realizada nas bases de dados do PubMed, ISI Web of Knowledge e Ovid, incluindo artigos publicados em inglês até 17 de maio de 2012. Esses deveriam relatar estudos observacionais humanos que apresentassem as palavras-chave: "aparelho ortodôntico fixo" e "colonização microbiológica"; ou "patógenos periodontais"; ou "*Streptococcus*"; ou "*Lactobacillus*"; ou "*Candida*"; ou "*Tannerella forsythia*"; ou "*Treponema denticola*"; ou "*Fusobacterium nucleatum*"; ou "*Actimomyces actinomycetemcomitans*"; ou "*Prevotella intermedia*"; ou "*Prevotella nigrescens*"; ou "*Porphyromonas gingivalis*". Artigos foram previamente selecionados pelo título e resumo. Artigos que atenderam aos critérios de inclusão foram analisados e classificados como de baixa, moderada ou alta qualidade metodológica. Uma nova lista de verificação detalhada para avaliação da qualidade foi desenvolvida, com base na informação necessária para a extração de dados aplicáveis para as revisões de literatura. O desenho do estudo, a amostra, o período de acompanhamento, coleta e métodos de análise microbiana, o tratamento estatístico, resultados e discussão foram avaliados.

**Resultados:**

a busca inicial encontrou 305 trabalhos. Em seguida, 33 artigos foram selecionados pelo título e resumo. Após a leitura do texto completo, apenas oito artigos preencheram os critérios de inclusão. Desses, quatro foram classificados como artigos com baixa qualidade metodológica e quatro como moderados. Os estudos de qualidade metodológica moderada foram incluídos na revisão sistemática.

**Conclusão:**

a literatura revelou evidência moderada de que a presença de aparelhos fixos exerce influência na quantidade e qualidade da microbiota bucal.

## INTRODUCTION

Scientific publications have demonstrated that the presence of fixed appliances in the oral cavity of orthodontic patients could alter the nature of dental plaque.^1^ The structure, metabolism and composition of dental plaque would change, leading to an increase in microbial population, especially *Streptococcus *and *Lactobacillus*.^1,7^

Some authors have observed that fixed appliances might hamper effective oral hygiene and cause high cariogenic challenge.^8,11^ Furthermore, based on the difficulty of maintaining oral hygiene, the subgingival microbiota may also be influenced by orthodontic appliances,^13,14^ since orthodontic accessories would favor bacterial plaque retention. These variables would possibly lead to pathogenic bacteria colonization, which are responsible for gingival inflammation, periodontal support destruction^13,14^ and changes in enamel surface.^7,8,11,12^

Published literature has shown that conditions favoring microbial colonization and establishment of *Streptococcus spp*, *Lactobacillus spp*, fungi and periodontal pathogens increase microbial population growth and plaque accumulation. Therefore, the following question arises: What is the real influence of orthodontic fixed appliances over balance of oral microbiota in orthodontic patients?^7,10,13-17^

Thus, the aim of this systematic review was to evaluate whether there is scientific evidence to support the hypothesis that the presence of orthodontic fixed appliances alters the composition of oral microbiota and, as a consequence, favors the development of caries and periodontal disease.

## MATERIAL AND METHODS

The search for articles was conducted in PubMed; ISI Web of Knowledge and Ovid databases. Articles published in English from 1945 to May 2012 were included. The following keywords were used in the preliminary search: "fixed orthodontic appliance" *AND* "microbiological colonization"; *OR* "periodontal pathogens"; *OR *"*Streptococcus*"*; OR* "*Lactobacillus*"; *OR* "*Candida*"; *OR* "*Tannerella forsythia*"; *OR *"*Treponema denticola*"; *OR* "*Fusobacterium nucleatum*"; *OR* "*Actimomyces actinomycetemcomitans*"; *OR* "*Prevotella intermedia*", *OR* "*Prevotella nigrescens*";* OR* "*Porphyromonas gingivalis*". As for the Ovid database, the keyword "periodontal pathogens" was replaced by "periodontal disease", since the former was not indexed. The search strategy and the flow of information through the different phases of the systematic review were established according to the PRISMA statement for systematic reviews and metanalysis.^18^ The articles were selected, evaluated and classified by two independent readers. The results of both readers were compared and eventual differences were solved by common accord.

Initially, all articles were selected by title and abstract. Should articles repeat in different databases, they were considered only once. The publications selected were essentially observational clinical studies conducted in humans. They were required to describe the microbial colonization in orthodontic fixed appliances in individuals submitted to corrective orthodontic treatment. Afterwards, inclusion and exclusion criteria were applied. The following inclusion criteria were applied: observational clinical studies in humans; presence of orthodontic fixed appliances placed onto the buccal tooth surface; standardization and training in oral hygiene; microbiological analysis of collected material. Conversely, the exclusion criteria were: absence of baseline investigation before appliances were placed; inclusion of patients with systemic diseases or under any condition that could influence oral microbiota or periodontal support tissues; antibiotic therapy three months before and during the study; use of mouth rinse during investigation; no standardization and training in oral hygiene; fixed or removable orthodontic appliances on the lingual dental surface. The articles that did not fulfil all the inclusion criteria in the abstract section, but did not meet any exclusion criteria were not excluded at this stage.

Subsequently, full text articles were read and those that met the inclusion and exclusion criteria were carefully analyzed and qualified according to their methodological aspects, as described in Table 1. A new detailed checklist for quality assessment was developed specifically for this review, based on the information required for data extraction applicable for reviews.^19^ The selected articles were finally classified according to the total score after qualification. Their methodological quality was classified as high (score 9 and 10), moderate (score from 6 to 8.9) or low (score from 0 to 5.9). Those classified as low were excluded. A hand search was performed to complement the previous searches, by which the references of the selected articles were analyzed.

**Table 1:** Methodological quality score.

Score protocol		Maximum score (10 points)
**1. Study design:** description of the study design		**0.2**
**2. Participants**		**1.2**
	**Sample standards:** participant's inclusion and exclusion criteria.	0.2
	**Sample characterization:** number and characteristic of participants.	0.2
	**Calculation of sample size**	0.6
	**Ethics:** evidence of ethical factors	0.2
**3. Study follow-up period**		**2.0**
	3 a 5 months	0.5
	≥ 6 months	1.5
	Month collections	0.5
**4. Collection methods**		**3.0**
	**Control of factors influencing collection:** collection under dry conditions, removal of supragingival plaque and debris	**-**
**5. Microbial analysis methods**		**3.0**
	Culture methods	1.0
	Molecular biology	1.5
	Mean standard deviation	0.5
**6. Statistical analysis:** adequate (indication of the test applied and significance level)		**0.2**
**7. Results:** adequate presentation of results (presentation of all proposed results; comparison between results considering time and microorganism; participant dropout with justification)		**0.2**
**8. Discussion:** consideration and possible explanation for the findings presented; comparison with previous published results		**0.2**

## RESULTS

A total of 305 titles and abstracts retrieved from the selected databases were screened. There were 136 from PubMed, 104 from Ovid and 65 from ISI Web of Knowledge. However, the duplicates were considered only once, thus totaling 250 articles.

Initially, the titles and abstracts not connected with the topic were excluded. Afterwards, the articles were selected according to the inclusion and exclusion criteria, thus totaling 33 articles. At this stage, all studies that presented at least one inclusion criterion in the abstract section, but presented none of the exclusion criteria, were kept. If any pre-selected article was inaccessible or not written in English, a direct request was made to the authors. After two months waiting for contact, 10 studies were excluded. Thus, 23 full texts were analyzed considering all selection criteria. The absence of any inclusion criteria at this stage determined the exclusion of 16 studies.

A hand search was performed with the references of the 7 remaining articles in order to screen additional publications not retrieved by the databases used. Thus, 23 new titles were retrieved. After reading the abstract, 12 articles were excluded. Subsequently, the texts of 11 articles were fully read and 10 other articles were also excluded. Therefore, 1 article obtained by hand search was added to the 7 previously selected articles.

**Figure 1:**
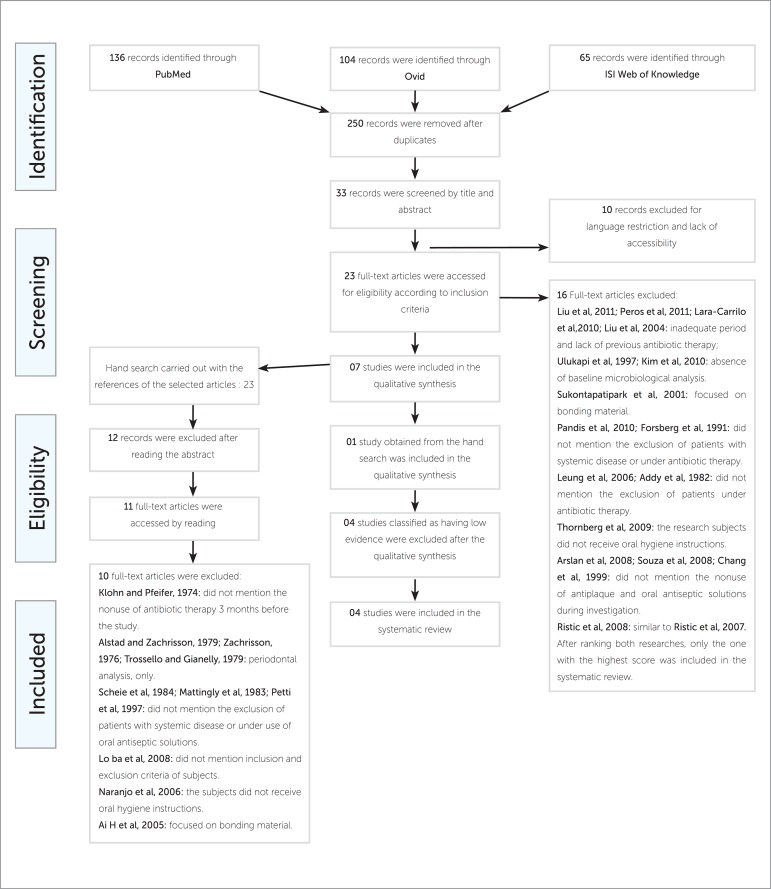
PRISMA Flow diagram of literature search.

Thus, the 8 selected articles were carefully read and ranked based on the quality assessment previously described, as shown in Table 2. After qualification, 4 articles were considered as having moderate scientific evidence whereas 4 were classified as having low scientific evidence. All articles classified as having low scientific evidence were excluded from this systematic review. Consequently, 4 studies were included (Fig 1). The distribution of articles is detailed in Table 3.

**Table 2:** Quality assessment.

Author/Year	Study design	Participants				Study follow-up period			Collection methods	Microbial analysis methods			Statistical analysis	Results	Discussion	Total points/ quality
		Sample Standards	Sample Characterization	Calculation of sample size	Ethics	3-5 month follow-up	≥ 6 month follow-up	1 month interval time	Control of factors influencing collection	Culture methods	Molecular biology	SEM				
Sinclair et al,^20^ 1987	0	0.2	0.2	0	0	0	1.5	0	3.0	1.0	0	0	0.2	0.2	0.2	6.5 / Moderate
Paolantonio et al,^23^ 1997	0	0.2	0.2	0	0	0	1.5	0	3.0	1.0	0	0	0.2	0.2	0.2	6.5 / Moderate
Paolantonio et al,^22^ 1999	0	0.2	0.2	0	0	1.0	0	0.5	3.0	1.0	0	0	0.2	0.2	0.2	6.0 / Moderate
Jordan et al,^27^ 2002	0	0.2	0.2	0	0	1.0	0	0.5	0	0	1.5	0	0	0	0.2	3.6 / Low
Hagg et al,^5^ 2004	0.2	0.2	0.2	0	0	1.0	0	0	0	1.0	0	0	0.2	0.2	0.2	3.2 / Low
Turkkahraman et al,^11^ 2005	0	0.2	0.2	0	0.2	0	0	0.5	0	1.0	0	0	0.2	0.2	0.2	2.7 / Low
Ristic et al,^21^ 2007	0.2	0.2	0.2	0	0.2	0	1.5	0	3.0	1.0	0	0	0.2	0.2	0.2	6.9 / Moderate
Andrucioli et al,^28^ 2012	0	0.5	0.5	0	0.2	0	0	0.5	0	0	1.5	0	0.2	0.2	0.2	3.8/ Low

**Table 3:** Characteristics of studies included in the review (detailed quality information).

Author/Year	Study design	Participant		Material collection time	Collection methods	Microbial analysis methods	Statistical analysis	Conclusion
		Sample Standards	General sample description	Total study time/ interval times	Control of factors influencing collection	Culture methods/ molecular biology/ SEM		
**Sinclair et al,^20^ 1987**	Not mentioned.	No history of orthodontic treatment, systemic disease or use of antibiotic therapy within the preceding 6 months; no fluoride rinse or gel was used before or during the study.	13 subjects, 8 males and 5 females, aged between 12 and 16 years old.	Collection at baseline and 1 year after placement of orthodontic appliance / Material from maxillary and mandibular central incisors, maxillary and mandibular right first permanent molars.	Plaque collected with 0.016' stainless steel orthodontic wire 20 mm in length inserted in the gingival cervices/ Under dry field conditions with cheek retractors, cotton rolls and aspiration.	Culture methods.	Two-way ANOVA for comparison between the sampling periods and Student's- t test for unmatched data.	Increase in the percentage of *Streptococci* and decrease in the percentage of *Aa* in the subgingival plaque; no increase in the percentage of potentially pathogenic Gram-negative organisms.
**Paolantonio et al,^23^ 1997**	Longitudinal study.	Subjects were not affected by systemic diseases, nor had taken antibiotics during the 3 months preceding each microbiological examination.	70 subjects, 27 males and 43 females, aged between 12 and 20 years old.	Collection before placement of fixed orthodontic appliance and 3 years after/Sampling was performed at mesio-buccal sites of 1^st^ molars and disto-buccal sites of lateral incisors.	Plaque samples: insertion of 3 sterile paper points at the deepest part of each gingival sulcus/ Removal of supragingival plaque by a sterile curette, gingival surface dried by gentle air flow.	Culture methods.	Mean % of Aa* in the total anaerobic flora from sampled sites was calculated for each individual; χ^2^ analysis to test the statistical significance of differences in the number of Aa* in orthodontic and control subjects.	The presence of orthodontic appliances significantly increases subgingival colonization by *Aa* among individuals presenting initial healthy periodontium.
**Paolantonio et al,^22^ 1999**	Not mentioned.	No loss of periodontal attachment, no systemic disease, no antibiotics taken during the 3 months before study or during it, and no mouthwash rinse.	24 subjects, 11 males and 13 females, aged between 18 and 22 years old.	Collection at baseline, 4, 8 and 12 weeks after bonding/ Material from mesiobuccal sites of the first molars and distobuccal sites of lateral incisors.	Plaque obtained by insertion of 3 sterile paper points at gingival sulcus/ Collected after removal of supragingival plaque with a sterile curette, gingival surface dried with air flow.	Culture methods.	Percentage of sites with positive results for Aa* mean values.	Placement of orthodontic appliance favors subgingival growth of Aa.
**Ristic et al,^21^ 2007**	Prospective longitudinal controlled study.	Indication for fixed orthodontic therapy; good initial general and periodontal health; lack of antibiotic therapy 3 months before and during the study and nonuse of antiplaque and oral antiseptic solutions during investigation.	32 subjects, 13 males and 19 females aged between 12 and 18 years old.	Collected just before placement of fixed appliances, and 1, 3 and 6 months after/ Collected from mesiovestibular points of subgingival sulcus of maxillary right first molar, maxillary left central incisor and maxillary left first premolar.	Subgingival plaque: inserting two sterile paper points (ISO 45)/ Collected in dry field conditions.	Culture methods.	Descriptive statistical measures, Student's- t test and chi-square test combined with McNemar test.	In adolescents, fixed orthodontic treatment increases the values of periodontal indices and growth of pathogenic and anaerobic bacteria.

* **Aa =** Actinobacillus actynomicetemcomitans

## DISCUSSION

Dental caries and periodontal diseases are recognized as consequences of inadequate oral hygiene during orthodontic treatment.^20^ Fixed appliances and rough-surfaced adhesives in the oral cavity create new retentive sites favorable to plaque accumulation and inflammatory response.^20,21^

Although new appliances as well as new bonding techniques and material have been developed, it has not yet been possible to decrease dental plaque retention.^20,21^ Therefore, plaque retention is considered a real problem in Corrective Orthodontics.^21^ Difficulties in maintaining oral hygiene around the appliances may result in hyperplastic marginal gingivitis which can advance to periodontitis.^20,22^

There is a consensus that oral microorganisms are the primary etiologic agents of periodontal diseases, and their different species are responsible for the different forms of the disease. Periodontal health is associated with supragingival gram-positive microbiota that consists mainly of diverse species of *Streptococci* and *Actinomyces*. They are also predominant in gingivitis, however, the amount of gram-negative bacteria, such as *Fusobacterium* and *Bacteroides,* increases. On the other hand, in periodontitis, the microflora is dominated by gram-negative facultative anaerobes, with increases spirochetes. The pathogenic potential of these microorganisms is related to their virulence and capacity to act in gingival tissues. Moreover, the virulence of bacteria depends on many factors, especially bacterial serotype and individual host susceptibility.^20,22^

In the retrieved articles, different microbiological studies were developed, revealing different aspects of supragingival plaque and important changes in the composition of subgingival plaque of orthodontic patients.^21^ These studies also reported the clinical implications of environmental disturbances caused by fixed appliances, considering the following as determinant factors of periodontal conditions: presence/absence of dental plaque, gingival bleeding index, pocket probing depth and attachment levels in orthodontic patients.^20-23^

Although the articles considered for this review reported that fixed orthodontic appliances influence the oral microbiota, it is relevant to point out that insufficient methodological information was provided.

Only two publications described the study design and the ethical aspects used for conducting the research.^5,21^ The diversified results observed can be attributed to the distinct methods applied. The different follow-up periods and collection times, as well as specificity and sensitivity of microbiological analysis were the major factors behind data diversity.^24,25^However, in all the studies included, the biological material was carefully collected so as to avoid interference from other sites in the oral cavity.^13,21-24,26,27^

The articles classified as having low methodological quality^5,11,27,28^ presented similarities that contributed to their low score and subsequent exclusion from the present review. Follow-up periods did not last for more than three months. Short-term researches are not suitable for investigating the influence of environmental changes in oral microbial colonization.^21,29^ These studies did not describe the control of influential factors that could interfere in biological material collection, a critical aspect in the research. Isolation of the area and previous removal of other biological material surrounding the collection site is essential.^13,21-24,26^

Despite the difficulty of isolating and maintaining microbial culture in laboratory, three articles used this method of investigation.^5,11,24,27^

One of them presented the results in an inappropriate manner.^24^ There was no comparison among results yielded at different times and no statistical treatment was applied. Andrucioli et al^28^ was the only one who provided an article that employed molecular biology techniques, however, it was excluded for conducting a one month follow-up. There was a lack of well-designed clinical trials using molecular biology techniques. It is worth noting that they are effective, but expensive methods that should be applied with better defined criteria.

The studies included in this systematic review were classified as having moderate methodological quality.^20,23^ Two of them^22,23^ were conducted by the same author, however, they presented different objectives, distinct samples and different follow-up periods. Both studies^22,23^ implemented similar methods to control the factors that could influence microbial collection. They conducted prior removal of supragingival plaque with a sterile curette and gingival surface drying with gentile air flow. Culture and subculture methods were selected for bacteriological analysis and definitive bacteria identification. Non-selective as well as selective culture media for *Actinobacillus actinomycetemcomitans* (*Aa)* were used. After incubation, the agar plates with selective culture media (trypticase soy-serum bacitracin vancomycin hydrochloride - TSBV) were examined and the colony-forming unit characteristics of *Aa* were subcultured. Definitive bacteria identification was carried out by means of the following methods: Gram-stain; nitrate reduction; production of catalase; urease and indole; growth on MacConkey agar and fermentation reactions to carbohydrates supplemented with the profiles of the enzymes used.

The first study,^23^ carried out in 1997, aimed to assess the occurrence of *Actinobacillus actinomycetemcomitans* (*Aa*) in 70 young patients and to determine if the presence of *Aa* at the baseline could influence the periodontal status 3 years after orthodontic treatment had been performed with fixed appliances. The clinical index for gingival bleeding revealed a significant decrease in the percentage of bleeding sites in orthodontic patients, although they presented more deteriorated gingival status. This could be explained by impaired plaque control caused by the presence of appliances. The mean percentage of positive plaque index remained stable among orthodontic patients during the study, although it was higher when compared with subjects without appliances. The study suggested that the presence of orthodontic appliances decreased the percentage of positive sites for *Aa* among treated patients (from 47.5% at baseline to 25.3% after 3 years). This fact may be associated with a successful host response, able to reduce and control *Aa* in the subgingival sites. The lack of association between subgingival *Aa *and inflammation in orthodontic patients can be due to the overgrowth of other bacterial species that could interfere in the virulence factors of *Aa*.

In 1999, Paolantonio et al^22^ aimed at establishing a direct relationship between orthodontic appliance placement and subgingival colonization by *Aa*. They also aimed at determining whether *Aa* colonization occurred only on teeth with appliances, or whether the presence of appliances could cause the isolation of *Aa* in teeth without appliances. A total of 24 individuals were observed from baseline to 1, 2 and 3 months after appliances had been placed. Results showed a significant increase in *Aa* until the first month of therapy and a stable mean of *Aa *prevalence between the first and the third month. *Aa* was isolated in 83.3% of sites after one month of therapy. Placement of orthodontic appliances was followed by worse gingival conditions with a tendency to bleeding and increase in plaque accumulation. The authors emphasized that the short period during which the study was conducted could have influenced the results, since another longitudinal 3-year study reported marked changes in the proportions of *Aa*.

Among all articles classified as having moderate methodological quality, only one described the study design and the ethical aspects considered for the research.^21^ The authors collected biological material at four different time intervals. There were baseline, one, three and six month observations of periodontopathic anaerobe colonizations: *Prevotlla intermedia* (*Pi*), *Aa*, *Porphyromonas gingivalis* (*Pg*) and *Fusobacterium nucleatum* (*Fn*). After isolation, bacteria were incubated for later semi-quantitative detection of anaerobe colonies using direct counting and density comparison. Subculturing, Gram-stain and identification tests of biochemical reactions were performed for accurate identification of bacterial species. According to this study, the maximum microbiological values were obtained three months after the beginning of the fixed orthodontic therapy. There was a decrease in microbiological parameters between the third and the sixth month after the appliances had been placed, which was explained based on the re-establishment of host-microorganism balance after the third month of therapy. The results of clinical parameters described an increase in the values of all clinical indices. Plaque index should be emphasized, since it increased until the third month of therapy, when the maximum score was recorded. Subsequently, the index decreased during the last months of investigation. The results confirmed the growth of periodontopathic bacteria in adolescents treated with fixed appliances, however, it was a transient condition.

In the last research included in this review,^20^ subgingival collection was performed before and one year after the appliances had been placed. The samples were collected using 0.016-in stainless steel orthodontic wire 20 mm in length inserted into gingival crevices. After preparation, each sample was plated onto five different selective media for subgingival bacteria. Plates were cultured in an anaerobic chamber at 37ºC for four days and the total count of viable bacteria was determined. Results did not show significant changes in subgingival microbiota during investigation, however, the percentage of the five types of bacteria was altered, especially *Streptococci* (17.5% of oral microbiota after one year). The increase in these bacteria is generally related to higher incidence of caries. On the other hand, there was a decrease in the percentage of *Actinomyces *(13.3% decrease in the total flora), and smaller reduction in *Fusobacterium* and *Bacteroides* species. Spirochetes had a statistically insignificant increase. These are potentially pathogenic gram-negative bacteria associated with periodontal disease. The insignificant increase in the number of gram-negative anaerobic bacteria was attributed to relatively good oral hygiene and consequently little increase in plaque accumulation. Therefore, no significant gingival pockets were formed. Clinical indices for periodontal health were used to complement the study. The supragingival index did not significantly change after one year of treatment, however, there was a correlation between orthodontic appliance use and plaque level for both time intervals. The gingival index showed an important increase for bonded teeth. The lack of correlation between gingival index and subgingival changes was also determined, which suggests that subgingival bacteria are not the only type directly responsible for gingival inflammation. The increase in *streptococci* in the supragingival plaque might have contributed to gingival inflammation. Therefore, the authors concluded that the effect of orthodontic fixed appliances on periodontal health should be further investigated and confirmed by studies conducted over longer periods of time.

Moreover, the hypothesis that orthodontic appliances create an ecologic environment favorable to qualitative alterations in the subgingival microbiota, and that orthodontic appliances were associated with poor oral hygiene and produced transitory gingival alterations incompatible with permanent damage to periodontal structures was accepted.^22^ It is extremely important to keep orthodontic patients under strict control of oral hygiene and plaque accumulation in order to favor rebalance between host and microorganism after appliance placement.^21^ Proper hygiene control leads to little increase in plaque accumulation in orthodontic patients, minimizing the possibility of tooth decalcification and development of inflammatory periodontal disease.^20^ Professional monitoring may motivate patients to maintain better self-performed plaque control and proper oral health.^23^

The results presented in this systematic review indicate that there is moderate scientific evidence that orthodontic fixed appliances influence the oral microbiota, given that no study was classified as presenting high methodological quality. Placement of appliances increases quantity and quality of oral microbiota. Apparently, this is a transitory effect which depends on oral hygiene. However, further well-designed studies conducted within longer periods of investigation, lower interval time between collections and more sensitive and specific microbiological analysis methods are needed to confirm the influence of orthodontic fixed appliances over oral microbiota. This strategy would confirm whether there is a critical period of time for the increase in microbiological colonization after the placement of orthodontic appliances. Therefore, orthodontists should reinforce the need for special oral hygiene control during orthodontic treatment.

## CONCLUSIONS

The literature revealed moderate evidence that the presence of fixed appliances influences the quantity and quality of oral microbiota. This might be a transitional effect that depends on oral hygiene control. The authors recommend that further investigations be developed on the influence of increasing quantity and quality of oral microbiota in the establishment of caries and periodontal diseases.

## References

[1] Campbel CHCT (2001). Alterações da microflora bucal em pacientes portadores de
aparelho ortodôntico fixo [tese].

[2] Balenseifen JW, Madonia JV (1970). Study of dental plaque in orthodontic patients. J Dent Res.

[3] Chin MYH, Busscher HJ, Evans R, Noar J, Pratten E (2006). Early biofilm formation and the effects of antimicrobial agents on
orthodontics bonding materials in a parallel plate flow chamber. Eur J Orthod.

[4] Friedman M, Harari D, Raz H, Golomb G, Brayer L (1985). Plaque Inhibition by sustained of Chlorhexidine from removable
appliances. J Dent Res.

[5] Hagg U, Kaveewatcharanont P, Samaranayake YH, Samaranayake LP (2004). The effect of fixed orthodontic appliances on the oral carriage of
candida species and enterobacteriaceae. Eur J Orthod.

[6] Olympio KPK, Bardal PAP, Henriques JFC, Bastos JRM (2006). Prevenção da cárie dentária e doença periodontal em
Ortodontia: uma necessidade imprescindível. Rev Dental Press Ortodon Ortop Facial.

[7] Sukontapatipark W, El-Agroudi MA, Selliseth NJ, Thunold K, Selvig KA (2001). Bacterial colonization associated with fixed orthodontic appliances. A
scanning electron microscopy study. Eur J Orthod.

[8] Brêtas SM, Macari S, Elias AM, Ito IY, Matsumoto MAN (2005). Effect of 0.44% fluoride gel on Streptococci mutans in relation to
elastomeric rings and steel ligatures in orthodontic patients. Am J Orthod Dentofacial Orthop.

[9] Chun M, Shim E, Kho E, Park K, Jung J, Kim J (2007). Surface modification of orthodontic wires photocatalytic titanium
oxide for its antiadherent and antibacterial properties. Angle Orthod.

[10] Souza RA, Magnani MBBA, Nouer DF, Silva CO, Klein MI, Sallum EA (2008). Periodontal and microbiologic evaluation of 2 methods of archwire
ligation: ligature wires and elastomeric rings. Am J Orthod Dentofacial Orthop.

[11] Tukkahraman H, Ozgur S, Bozkurt FY, Yetkin Z, Kaya S, Onal S (2005). Archwire ligation techniques, microbial colonization, and periodontal
status in orthodontically treated patients. Angle Orthod.

[12] Gorelick L, Geiber AM, Gwinnett AJ (1982). Incidence of white spot formation after bonding and
banding. Am J Orthod Dentofacial Orthop.

[13] Naranjo AA, Triviño ML, Jaramillo A, Betancourth M, Botero JE (2006). Changes in the subgingival microbiota and periodontal parameters
before and 3 months after bracket placement. Am J Orthod Dentofacial Orthop.

[14] Thornberg MJ, Riolo CS, Bayirli B, Riolo ML, Van Tubergen EA, Kulbersh R (2009). Periodontal pathogen levels in adolescents before, during and after
fixed orthodontic appliance therapy. Am J Orthod Dentofacial Orthop.

[15] Bloom RH, Brown LRJR (1964). Study of the effects of orthodontic appliances on the oral
microflora. Oral Surg Oral Med Oral Pathol.

[16] Uetenabaro T (1980). Acúmulo de placa bacteriana em pacientes portadores de colagem
direta e anéis convencionais [tese].

[17] Walker MP, Ries D, Kula K, Ellis M, Fricke B (2007). Mechanical properties and surface characterization of beta titanium
and stainless steel orthodontic wire following topical fluoride
treatment. Angle Orthod.

[18] Moher D, Liberati A, Tetzlaff J, Altman DG, The PRISMA Group (2009). Preferred Reporting Items for Systematic Reviews and Meta-Analyses:
The PRISMA Statement. PLoS Med.

[19] Centre for Reviews and Dissemination (2009). Systematic reviews: CRD's guidance for undertaking reviews in health
care.

[20] Sinclair M, Berry CW, Bennet CL, Israelson H (1987). Changes in gingival and gingival flora with bonding and
banding. Angle Orthod.

[21] Ristic M, Vlahovic SM, Sasic M, Zelic O (2007). Clinical and microbiological effects of fixed orthodontic appliances
on periodontal tissues in adolescents. Orthod Craniofac Res.

[22] Paolantonio M, Festa F, Di Placido G, D' Attilio M, Catamo G, Piccolomini R (1999). Site-specific subgingival colonization by Actinobacillus
actinomycetencomitans in orthodontic patients. Am J Orthod Dentofacial Orthop.

[23] Paolantonio M, Pedrazzoli V, Di Muro C, di Placido G, Picciani C, Catamo G (1997). Clinical significance of actinobacillus actinomycetemcomitans in young
individuals during orthodontic treatment. J Clin Periodontol.

[24] Morikawa M, Chiba T, Tomii N, Sato S, Takahashi Y, Konishi K (2008). Comparative analysis of putative periodontopathic bacteria by
Multiplex Polymerase Chain Reaction. J Periodont Res.

[25] Sakamoto M, Takeuchi Y, Umeda M, Ishikawa I, Benno Y (2001). Rapid detection and quantification of five periodontopathic bacteria
by Real-Time PCR. Microbiol Immunol.

[26] Jervoe-Storm PM, Alahdab H, Koltzscher M, Fimmers R, Jepsen S (2007). Comparison of curet and paper point sampling of subgingival bacteria
as analyzed by real-time polymerase chain reaction. J Periodontol.

[27] Jordan C, Leblanc DJ (2002). Influences of orthodontic appliances on oral populations of mutans
streptococci. Oral Microbiol Immunol.

[28] Andrucioli MCD, Nelson-Filho P, Matsumoto MAN, Saraiva MCP, Feres M, Figueiredo LC (2012). Molecular detection of in-vivo microbial contamination of metallic
orthodontic brackets by checkerboard DNA-DNA hybridization. Am J Orthod Dentofacial Orthop.

[29] Heuer W, Elter C, Demling A, Neumann A, Suerbaum S, Hannig M (2007). Analysis of early biofilm formation on oral implants in
man. J Oral Rehabil.

